# Hybrid gelatin/oxidized chondroitin sulfate hydrogels incorporating bioactive glass nanoparticles with enhanced mechanical properties, mineralization, and osteogenic differentiation

**DOI:** 10.1016/j.bioactmat.2020.09.012

**Published:** 2020-10-06

**Authors:** Lei Zhou, Lei Fan, Feng-Miao Zhang, Yuhe Jiang, Min Cai, Cong Dai, Yi-An Luo, Ling-Jie Tu, Zheng-Nan Zhou, Xiao-Jun Li, Cheng-Yun Ning, Kai Zheng, Aldo R. Boccaccini, Guo-Xin Tan

**Affiliations:** a Institute of Chemical Engineering and Light Industry, Guangdong University of Technology, Guangzhou, 510006, China; b Institute of Biomaterials, Department of Materials Science and Engineering, University of Erlangen-Nuremberg, Cauerstrasse 6, 91058, Erlangen, Germany; c School of Materials Science and Engineering, South China University of Technology, Guangzhou, 510641, China; d College of Arts and Sciences, Stony Brook University, 100 Nicolls Road, Stony Brook, NY, 11794, United States; e Department of Prosthodontics, Hospital of Stomatology, Guanghua School of Stomatology, Sun Yat-sen University, Guangzhou, 510055, China

**Keywords:** Hydrogels, Bioactive glasses, Biopolymers, Hybrids, Bone regeneration

## Abstract

Biopolymer based hydrogels are characteristic of their biocompatibility and capability of mimicking extracellular matrix structure to support cellular behavior. However, these hydrogels suffer from low mechanical properties, uncontrolled degradation, and insufficient osteogenic activity, which limits their applications in bone regeneration. In this study, we developed hybrid gelatin (Gel)/oxidized chondroitin sulfate (OCS) hydrogels that incorporated mesoporous bioactive glass nanoparticles (MBGNs) as bioactive fillers for bone regeneration. Gel-OCS hydrogels could be self-crosslinked in situ under physiological conditions in the presence of borax. The incorporation of MBGNs enhanced the crosslinking and accelerated the gelation. The gelation time decreased with increasing the concentration of MBGNs added. Incorporation of MBGNs in the hydrogels significantly improved the mechanical properties in terms of enhanced storage modulus and compressive strength. The injectability of the hydrogels was not significantly affected by the MBGN incorporation. Also, the proliferation and osteogenic differentiation of rat bone marrow mesenchymal stem cells in vitro and rat cranial defect restoration in vivo were significantly promoted by the hydrogels in the presence of MBGNs. The hybrid Gel-OCS/MBGN hydrogels show promising potential as injectable biomaterials or scaffolds for bone regeneration/repair applications given their tunable degradation and gelation behavior as well as favorable mechanical behavior and osteogenic activities.

## Highlights

• In situ self-crosslinking of hybrid Gel-OCS/MBGN hydrogels.
• Hybrid hydrogels are porous, injectable and bioadhesive.
• Incorporation of MBGNs enhances mechanical and mineralization properties of hydrogels.
• Osteogenic differentiation of BMSCs enhanced after incorporating MBGNs into hydrogels.
• The presence of MBGNs enhances in vivo rat cranial defect restoration.

## Graphical abstract

**Figure:**
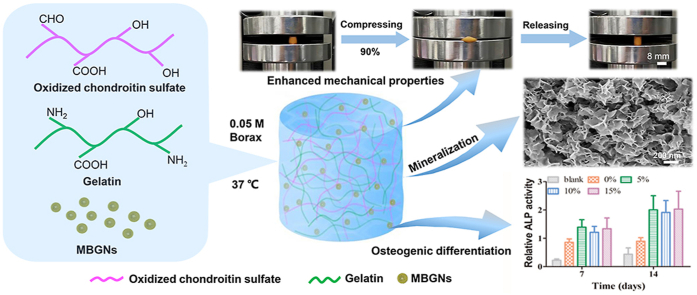

## 1 Introduction

Hydrogels can mimic the structure of the extracellular matrix (ECM) that provides a 3D environment for cell adhesion, ingrowth, and proliferation as well as promoting the transfer of soluble nutrients and metabolic waste [1,2]. Owing to their unique properties, hydrogels have been widely applied in various biomedical applications including tissue engineering and nanomedicine [1,3]. Particularly, biopolymer (e.g., proteins, polysaccharide) based hydrogels represent promising biomaterials for tissue regeneration applications due to their superior biocompatibility, abundant surface groups for functionalization, and the ability to immobilize biomolecules (e.g., growth factors) [1,4]. Chondroitin sulfate (CS) is a glycosaminoglycan that can be found in the non-collagenous ECM of human bone. CS has shown the potential to improve bone regeneration considering its ability to increase the efficacy of arrangement of certain growth factors involved in bone regeneration [5]. Gelatin, a water-soluble biocompatible biopolymer, contains a large number of glycine, proline and 4-hydroxyproline residues that facilitate cell adhesion and biomolecule deposition. It has a sol-gel transition temperature at around 30 °C and can naturally form hydrogels at relatively low temperatures, but it should be crosslinked to avoid fast dissolution at physiological temperature (37 °C). Biopolymer based hydrogels still suffer from weak mechanical strength, relatively fast degradation, and insufficient osteogenic and angiogenic activities [1,6]. These limitations impair the successful applications of biopolymer-based hydrogels in tissue regeneration, particularly in bone regeneration/repair.

Many efforts have been dedicated to modulating degradation behavior and mechanical properties as well as promoting biological functionalities of biopolymer-based hydrogels, including control of crosslinking [7], surface modification [8], and incorporation of bioactive fillers [9]. Among these strategies, the introduction of bioactive nanoparticles can actively regulate the mechanical behavior of hydrogels and their degradation kinetics as well as providing novel functionalities (e.g., osteogenic and antibacterial activities) [9,10]. Various types of nanoparticles have been applied as bioactive fillers to enhance the properties of hydrogels, such as silica nanoparticles [11] and silicon nanoparticles [12]. Bioactive glass nanoparticles (BGNs) are attracting increasing attention as building blocks for developing nanocomposites and hybrids, considering their controllable particle size/shape, bioreactivity, and degradation rate that can lead to superior biocompatibility, bioactivity, osteogenic and angiogenic activities [13,14]. In particular, incorporation of BGNs into biopolymer-based hydrogels can enhance the mechanical properties, osteogenic and angiogenic activities [15,16]. For example, Cu-doped BGNs were incorporated into chitosan/silk fibroin based hydrogels, which enhanced osteogenic and angiogenic activities of the hydrogel as well as the in vivo bone regeneration capability [16]. Moreover, mesoporous BGNs (MBGNs), due to the high specific surface area and porosity, can interact with polymeric matrices and biomolecules to a greater extent in comparison to nonporous BGNs, which may lead to enhanced mechanical reinforcement and biological activities. For example, Xin et al. [17] combined amine-functionalized MBGNs with methacrylate gelatin (GelMA) hydrogel, obtaining enhanced mechanical properties, as well as angiogenesis and osteogenesis effects.

In this study, we aimed to enhance the mechanical and biological properties of gelatin (Gel)/oxidized chondroitin sulfate (OCS) hydrogels by incorporating MBGNs as bioactive fillers. Synthesis and characterization of Gel-OCS hydrogels have been reported [18]. However, the application of Gel-OCS hydrogel in bone regeneration is rarely focused, even though this type of hydrogel possesses biocompatibility and tunable degradation rate. Thus, we firstly investigated the in situ gelation behavior of Gel-OCS hydrogels under mild conditions in the presence of borax. MBGNs were then incorporated into the Gel-OCS hydrogels at different concentrations. The effects of MBGN incorporation on the gelation behavior, mechanical properties and biological properties of the hydrogels were comprehensively investigated.

## 2 Materials and methods

### 2.1 Materials

Chondroitin 4-sulfate sodium salt (CS, cell culture, ≥ 85%), gelatin (type B, gel strength ~100 g Bloom), sodium tetraborate decahydrate (Na_2_B_4_O_7_·10H_2_O, GR, 99.5%), ammonia solution (AR, 25–28%), borax, sodium periodate (AR, ≥99.5%) and calcein (AM) were purchased from Aladdin (Shanghai, China). Tetraethyl orthosilicate (TEOS, AR, 98%) was purchased from Sigma-Aldrich (St. Louis, USA). Hexadecyl trimethyl ammonium bromide (CTAB, AR, ≥99%) was purchased from Shanghai Sinopharm Chemical Reagent Co. Ltd. Calcium nitrate (CN, AR, 99%) was provided by Tianjin Fuchen Chemical Reagent Factory. Ethyl acetate (EA, AR, ≥99.5%) and absolute ethanol (AR, ≥99.7%) were purchased from Guangdong Guanghua Technology Co. Ltd. Rat bone marrow mesenchymal stem cells (BMSCs) were provided by the Stem Cell Bank, Chinese Academy of Sciences. Dulbecco's modified Eagle medium (DMEM) and Fetal Bovine Serum (FBS) were purchased from HyClone, USA. Actin-Tracker Green, 4′, 6-diamidino-2-phenylindole (DAPI), Propidium Iodide (PI), Cell Counting Kit-8 (CCK-8) and Alkaline Phosphatase Assay Kit (ALP) were purchased from Beyotime Biotechnology.

### 2.2 Synthesis of oxidized chondroitin sulfate (OCS)

OCS was synthesized using a method reported previously [19]. Briefly, 1.25 g of CS was dissolved in 20 mL of distilled water and stirred at 4 °C. After CS was completely dissolved, 1.93 g of sodium periodate was added to the CS solution and allowed for a reaction for 6 h in a dark environment. The molar ratio of CS to sodium periodate was set as 1:1 for oxidation. Finally, the above solution was placed in a dialysis bag (3500 MW cut-off) and dialyzed against distilled water (2 L) for 24 h at room temperature. Water was exchanged every 6 h. After that, the dialyzed solution was poured into a 50 mL centrifuge tube, frozen at −20 °C for 24 h, and then lyophilized in a freeze dryer (FD-10, China) for 7 days to obtain the OCS powders.

### 2.3 Synthesis of MBGNs

MBGNs were synthesized as described in previous studies [20,21]. Briefly, 2.80 g CTAB was dissolved in 132 mL deionized water under stirring at 35 °C. When the CTAB was completely dissolved, 40 mL EA was added. After stirring for 30 min, 28 mL ammonia solution (1 mol/L) was added. Then the mixture was stirred for 15 min, followed by the addition of 14.40 mL TEOS. After 30 min of stirring, 6.52 g CN was added. The above mixture was further stirred for 4 h. The solution gradually became milky white due to the formation of colloids. The colloidal particles were collected by centrifugation at 8000 rad/s and washed three times with water and three times with ethanol. The collected precipitates were dried at 60 °C for 24 h and then ground into fine powders with a mortar. Finally, MBGNs were obtained by heating to 700 °C for 3 h at a heating rate of 2 °C/min to remove organics and nitrates.

### 2.4 Preparation of hybrid Gel-OCS/MBGNs hydrogels

Briefly, the OCS powders were dissolved in borax aqueous solution (0.05 M) to form the OCS solution (10% w/v). MBGNs were then added into the OCS solution at different concentrations under ultrasonic dispersion for 10 min at room temperature to obtain the OCS solution containing MBGNs. Gelatin was dissolved in borax aqueous solution (0.05 M) at 60 °C to form gelatin solution (30% w/v). Then, OCS/MBGN mixtures and gelatin solution were uniformly mixed at a volume ratio of 1:1 and poured into a mold to form hybrid Gel-OCS/MBGN hydrogels at 37 °C. The final concentrations of MBGNs in the resulting hydrogels were 0, 5, 10, and 15% (w/w) that were determined according to the weight ratio of MBGNs/(MBGNs + OCS + Gel).

### 2.5 Material characterization

#### 2.5.1 Characterization of OCS

CS and OCS were separately dissolved in deuterium oxide (D_2_O, 99.9%, Adamas-beta) for nuclear magnetic resonance (NMR) analysis (AVANCE III HD 400, Bruker) with tetramethylsilane (TMS) as the internal standard at 25 °C. The chemical structure and functional groups of MBGNs and OCS were examined by Fourier transform infrared spectroscopy (FTIR) (Nicolet 6700, Thermo-Fisher, USA) in transmission mode at a 4 cm^−1^ resolution and 16 scans with a wavelength range from 400 to 4000 cm^−1^.

#### 2.5.2 Field emission scanning electron microscopy (FE-SEM)

The morphology of MBGNs and Gel-OCS/MBGN composite hydrogels was examined by FE-SEM (Ultra 55, Carl Zeiss AG, Germany). Before the characterization, MBGNs were ultrasonically dispersed in ethanol and then dropped on a silicon wafer. The hydrogel samples were lyophilized for 3 days before SEM observation. All samples were sputter-coated with gold (SC7620, Quorum Technologies, UK) for 60 s. During the SEM observation, MBGNs and Gel-OCS/MBGN composite hydrogels were examined by energy dispersive spectroscopy (EDS).

#### 2.5.3 X-ray diffraction (XRD) and FTIR

MBGNs and the mineralization layers of hydrogels were analyzed by XRD (Ultima III, Japan) at the generator voltage of 40 kV and the tube current of 40 mV. The scan speed was 2°/min and 2θ range was 10–80°. The chemical structure and functional groups of the composite hydrogels were examined by FTIR in transmission mode at a 4 cm^−1^ resolution and 16 scans with a wavelength range from 500 to 4000 cm^−1^. The Gel-OCS/MBGN hydrogels were analyzed after lyophilization.

#### 2.5.4 Rheological and dynamic mechanical thermal analysis

An oscillation time scanning experiment was performed to record the storage (elastic) modulus (G′) and the loss modulus (G") to determine the gelation time of the hydrogels. The oscillation frequency was set to 1 Hz and a shear strain of 5% was applied.

Rheological properties of the hybrid hydrogels were evaluated using a rotary rheometer (Physica MCR301, Anton Paar). The crosslinked hydrogels (Φ25 mm × 2 mm) were placed on a sample stage and tested with a 25 mm diameter flatbed device at 37 °C. The storage modulus (G′) and loss modulus (G") were obtained from the frequency–modulus curves between 0.1 and 100 rad/s at a 5.0% strain amplitude.

The compressive strength of saturated hybrid hydrogels was investigated using a dynamic mechanical thermal analysis (DMA, Q800, TA Instruments, USA) with a pressurization rate of 3 N/min at 25 °C. The compressive modulus of the crosslinked hydrogels (Φ10 mm × 5 mm) was calculated from the linear segment of the stress-strain curves obtained from the DMA test.

### 2.6 *In vitro* mineralization and degradation

The mineralization test of the hydrogels was performed in simulated body fluid (SBF) according to the protocol proposed by Kokubo et al. [22]. Briefly, the crosslinked hydrogel (Φ8 mm x 2 mm) were soaked 10 mL SBF in centrifuge tubes in a shaker at 37 °C. The SBF was exchanged every two days. The samples were taken out from the SBF solution after 7 days of incubation and rinsed with deionized water to remove the excess SBF. After lyophilization, the samples were characterized by SEM-EDS, FTIR, and XRD as described above.

The mass loss of the hydrogels was measured in PBS (pH 7.4) to evaluate the in vitro degradation. Briefly, approximately 1 mL of freshly prepared hydrogel was added into 3 mL PBS at 37 °C. PBS was removed and the mass was recorded every day until the complete degradation of the hydrogels. The mass loss was calculated using Eqn (1):

(1) Mass loss (%)=(M1−M2)/M1×100%

where M1 and M2 are the mass of the hydrogels before and after soaking in PBS, respectively.

The changes in the pH value of PBS containing the hydrogels were measured.

### 2.7 *In vitro* cytotoxicity, proliferation, and adhesion

Rat bone marrow mesenchymal stem cells (BMSCs) were used for the in vitro assay. The BMSCs were cultured in DMEM supplemented with 10% FBS, 2 mM l-glutamine, and 1% penicillin-streptomycin in a 5% CO_2_ incubator at 37 °C. BMSCs were seeded on the surface of the sterilized hydrogels (Φ 8 mm × 1 mm, sterilization in 75% ethanol) at a density of 1 × 10^4^ cells per well in 48-well plates. After 48 h of incubation in a 5% CO_2_ incubator at 37 °C, the cells were fixed with 4% glutaraldehyde and then stained with Actin-Trakcer Green and DAPI staining solution. The cell adhesion and spreading were observed under laser confocal microscopy (Leica TCS SP5, Germany Leica Instrument Co., Ltd.). Cell proliferation was assessed on the hydrogels using the CCK-8 assay after 1 day, 4 and 7 days of culture following the manufacturer's instruction. At predetermined time points, the optical density (OD) value at a wavelength of 450 nm was measured (n = 5) by a Multiskan FC type plate reader (Semerce Aerospace Instruments Co., Ltd.). The cell viability on the hydrogels after 1 day of culture was assessed using the calcein-AM/propidium iodide-PI live/dead affinity assay kit and using laser confocal microscopy. The cell spreading areas were measured using ImageJ software (National Institutes of Health, Bethesda, MD, USA).

### 2.8 Osteogenic differentiation

To induce BMSCs into osteogenic differentiation, cells were cultured on the surface of the hydrogels in osteogenic differentiation medium (supplied with 10 mM/L β-glycerophosphate, 0.1 mM/L dexamethasone and 0.05 mM/L ascorbic acid) for 7 and 14 days. The differentiation medium was changed every 3 days.

### 2.9 Immunostaining

After 14 days of culture, attached cells were washed three times with PBS and fixed with 4% paraformaldehyde for 30 min at room temperature. Cells were incubated in 0.1% TritonX-100 in PBS for 15 min at room temperature and blocked with 1% BSA in PBS for 2 h at room temperature. The fixed cells were incubated overnight at 4 °C in solutions of primary antibodies (anti-OPN (ab8448, 1:200; Abcam) and anti-RUNX2 (ab23981, 1:200; Abcam) in PBS containing 5% BSA. After being washed three times with PBS, the samples were exposed to secondary goat anti-mouse IgG conjugated with Alexa-Fluor 594 (1:500; Abcam). After washing, cell nuclei were stained by Hoechst. The images were captured using a Leica TCS SP8 confocal microscope.

### 2.10 Alkaline phosphatase (ALP) staining

Alkaline phosphatase (ALP) activity, an indicator of early osteogenesis, of BMSCs cultured on the hydrogels was evaluated using an ALP assay kit according to the manufacturer's instruction. The lysates obtained by digestion of BMSC cells were reacted with p-nitrophenyl phosphate (p-NPP) on day 7 after culture. The absorbance was measured at a wavelength of 405 nm using a Multiskan FC type microplate reader to indicate the amount of ALP.

### 2.11 Alizarin Red S staining

After cultured in osteogenic medium for 14 days, cells were fixed in 4% paraformaldehyde for 30 min at room temperature, and then stained in 1% (w/v) Alizarin Red S (pH 4.2, Sigma-Aldrich) for 1 h. After the samples were washed with PBS three times, photos were taken. The Alizarin red dye was subsequently dissolved in 5% cetylpyridinum chloride (Sigma-Aldrich) in 10 mM sodium phosphate at room temperature. The absorption value at 570 nm was then recorded using a microplate reader (Thermo Multiskan FC).

### 2.12 Real-time (RT)- PCR experiments

Total RNA was extracted from the cells cultured on each sample by the Trizol reagent after 14 days of culture. The RNA was then reverse transcribed into cDNA using a reverse transcription kit (Takara, Japan). The RT-PCR was performed using the MaximaTM SYBR Green/ROX qPCR Master Mix (Thermo). All reactions were carried out in triplicate. The primers sequences are listed in Table S1.

### 2.13 Western blot

To extract proteins, cells were lysed with the protein lysate containing protease inhibitor (Thermo Fisher) and phosphatase inhibitor. The total protein concentration was determined with the BCA kit (Beyotime). The protein suspension was transferred to a polyvinylidene fluoride membrane. Membranes were subsequently incubated with primary antibodies (Anti-OPN, anti-OCN) at 4 °C overnight and the corresponding secondary antibody for 1 h. The chemiluminescence (ECL) kit (Thermo Fisher) was used to visualize the bands.

### 2.14 Animals and surgical procedures

Two critical-sized bone defects (5 mm diameter) were prepared in each rat at the center of each skull using a mini-cranial drill. After the defects were stabilized, the hydrogel was injected to fill the defect sites. 6 weeks after hydrogel implantation, rats were euthanized, and skulls were obtained. Bone regeneration was evaluated after surgery and material implantation using micro-CT and histological analysis. Animal experiment protocols were approved by the Laboratory Animal Ethics Committee of South China University of Technology.

### 2.15 Microcomputed tomography (Micro-CT) and histological analysis

The calvaria samples were harvested and fixed with 4% paraformaldehyde. The specimens were subsequently examined with a micro-CT instrument (SCANCO mCT50; Switzerland) at a voltage of 70 kV, a current of 200 μA, and a resolution of 20 μm. 3D reconstruction was performed using microtomographic analysis software. The quantification of the newly regenerated bone volume was calculated by software (Materialise Mimics Research 19) using the threshold of Hu value from 600 to 1300. After micro-CT analysis, the tissue samples were embedded in 10% neutral buffered formalin, decalcified and dehydrated. Tissue sections with 10 μm thickness were sliced by a rotary microtome (RM2255, Leica, Germany), and stained with hematoxylin and eosin (H&E) and Masson's trichrome. Images were captured with a microscope (Nikon E800 upright microscope).

### 2.16 Statistical analysis

All quantitative data are presented as mean ± SD and the quantitative experiments were carried out at least in quadruplicate. Differences between the experimental and the control group were analyzed by one-way analysis of variance, and *p < 0.05 was considered as statistically significant. Statistical analysis and data processing were performed using GraphPad Prism 5 and Origin Pro. 8.

## 3 Results and discussion

### 3.1 Synthesis and characterization of OCS and MBGNs

OCS was synthesized by using sodium periodate to oxidize CS as reported in the literature [18]. The process is schematically illustrated in Fig. S1a. Nuclear magnetic resonance and FTIR results confirmed the oxidization of CS. ^1^H-NMR spectra (Fig. S1b) show that a characteristic aldehyde-based peak located at 8.28 ppm was only observed in the spectrum of CS after the oxidation. Moreover, the FTIR spectrum of OCS (Fig. S1c) shows a new band located at 1716 cm^−1^ that could be assigned to the stretching vibration of the aldehyde group formed [18]. However, this band was not observed in the spectrum of CS, indicating that the hydroxyl groups of CS were oxidized to aldehyde groups. MBGNs were synthesized using a microemulsion assisted sol-gel method [13]. SEM image of MBGNs (Fig. S2a) shows the sphere-like shape of the particles with a particle size of ~100 nm. The morphology of MBGNs was in good agreement with that of mesoporous silicate nanoparticles synthesized using similar methods [20,23]. The mesoporous structure, large specific surface area and pore volume of MBGNs have been confirmed in our previous study [20]. The morphological characteristics of MBGNs were expected to enhance the dispersion in polymeric matrices and interactions with polymeric molecules towards a more integrated structure [13,24]. The chemical composition of MBGNs has been determined to be ~85SiO_2_–15CaO (mol%) and proved to be non-cytotoxic in our previous study [20]. Here the EDS spectrum of MBGNs (inserted in Fig. S2b) confirms the presence of calcium (Ca) and silicon (Si) in the particles. The XRD pattern of MBGNs (Fig. S2c) verified the amorphous structure of the nanoparticles, as only the characteristic broad hump of amorphous silicate phase in the range of 2θ = 20–30° could be observed. FTIR spectrum (Fig. S2d) shows characteristic bands of silicate nanoparticles located at ~440 and 812 cm^−1^ that could be assigned to the bending and symmetric stretching vibration of Si–O–Si, respectively. The broadband located between 1300 and 1000 cm^−1^ could be assigned to asymmetric Si–O–Si (bridging bonds) and Si–O-(non-bridging bonds) vibrations [25]. Taking together, the results confirmed the successful synthesis of OCS and MBGNs that were used as building blocks to develop hybrid hydrogels.

### 3.2 Incorporation of MBGNs facilitates gelation of Gel-OCS hydrogels

**Fig. 1:**
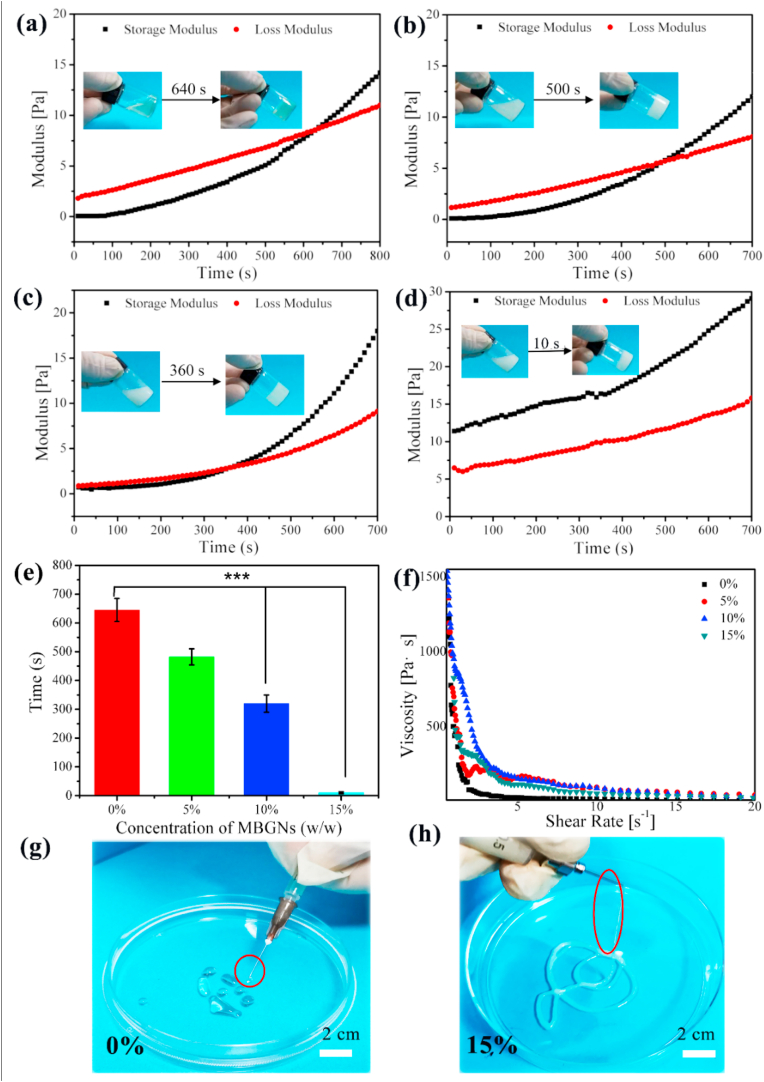
Storage modulus (G′) and loss modulus (G’’) of hybrid Gel-OCS/MBGN hydrogels change overtime at 37 °C. The hydrogels incorporating different concentrations of MBGNs: (a) 0%, (b) 5%, (c) 10%, (d) 15%. (e) Gel time of Gel-OCS/MBGN hydrogel. (f) Shear-thinning behavior of Gel-OCS/MBGN hydrogels. (g–h) Injection of Gel-OCS/MBGN hydrogels (0% and 15%) from the syringe.

To evaluate the gelation time of the Gel-OCS and Gel-OCS/MBGN hydrogels, the time sweep rheology analysis was performed at the physiological temperature 37 °C. The gelation of hydrogels was determined when the storage modulus (G′) value of hydrogels was greater than the loss modulus (G’’) [26]. For the Gel-OCS hydrogel, G′ was greater G’’ after 640 s of reaction, indicating the gelation of hydrogels (Fig. 1a). After the addition of MBGNs, the occurrence of the crosslinking between OCS and gelatin was not prevented (Fig. 1), however, the gelation time was dramatically affected. As can be seen in Fig. 1, the gelation time was shortened with increasing the concentration of MBGNs added (Fig. 1b–d) as determined by the crossover of G′ and G’’ values. When the concentration of MBGNs added reached 15%, the gelation (10 s) was significantly faster than those of other hydrogels (640 s for 0%, 500 s for 5%, and 360 s for 10% Gel-OCS/MBGN hydrogels). In comparison to Gel/OCS hydrogel, the gelation time was observed to be ~1.28, 1.78, and 64 times faster for the hybrid hydrogels incorporating 5, 10, and 15% (w/w) MBGNs, respectively. Notably, the Gel-OCS/MBGN (15%) composition could rapidly form hydrogels as soon as the homogenous mixture of OCS/MBGN and gelatin, even before the rheology test as indicated by the larger G′ than G’’ in the analysis (Fig. 1d).

CS is readily water-soluble and usually needs chemical crosslinking to form stable hydrogels for in vitro and in vivo applications. Gelatin degrades fast at the physiological temperature (37 °C) which limits its applications when relatively long-term stability is required (e.g., bone scaffolds). Crosslinking (either chemical or enzymatic) treatments are usually required to reduce the degradability of these biopolymers at the physiological temperature [1,27,28]. CS has been blended or hybridized with other biopolymers (e.g., collagen, chitosan) to enhance the stability and to reduce degradability [29,30]. It has been known that OCS can interact with gelatin to form hydrogels predominantly based on the Schiff's base reaction between the ε-amino groups of lysine or hydroxylysine side groups of gelatin and the aldehyde groups in OCS [18]. To facilitate the Schiff's base reaction, borax was added in the system in this study, which could interact with OCS via borate-diol complexation, leading to the branching of OCS molecules through the physical association of OCS chains while remaining soluble state [31]. Borax has been widely used to facilitate the crosslinking between polysaccharides (e.g., oxidized alginate) and gelatin [31,32], as hydroxyl groups of polysaccharides can serve as ligands for complexation with B(OH)_4_^-^ and B(OH)_3_ that are from dissociation in the aqueous medium [33], by which more polymer chains could be linked to other chains at multiple points, resulting in covalent bonding (Schiff's base formation) to a greater extent. Therefore, OCS could be crosslinked with gelatin, which led to the formation of in situ self-crosslinked hydrogels without the addition of chemical crosslinking agents that could induce potential cytotoxicity. Also, it can be observed that the gap between the storage modulus (G′) value and loss modulus (G’’) curves (Fig. 1) of the hydrogels became significantly smaller with the incorporation of MBGNs, suggesting that the presence of MBGNs enhanced the elasticity of hydrogels (Fig. 1e).

**Fig. 2:**
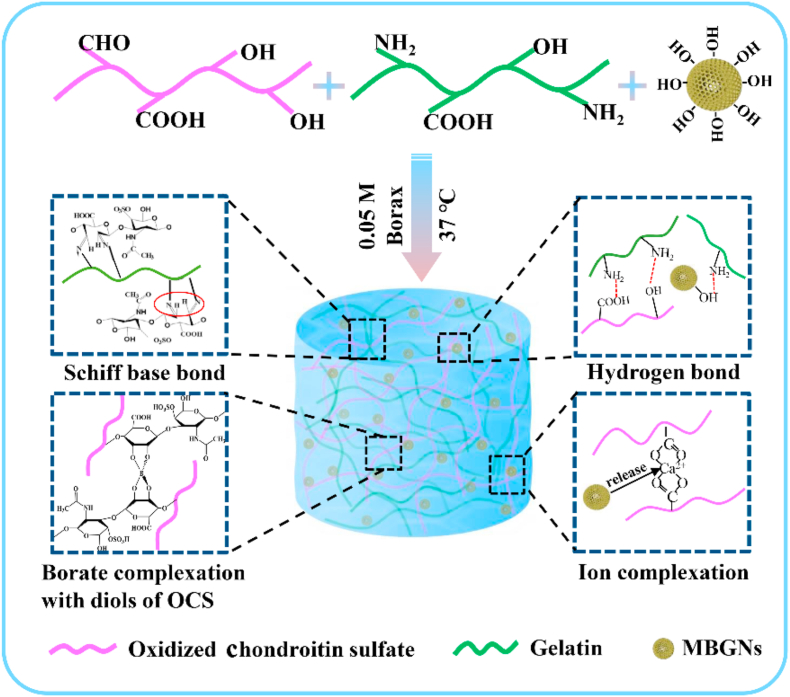
Schematically illustration of mechanisms involved in the gelation of hybrid Gel-OCS/MBGN hydrogels.

Several interactions contributed to the accelerated gelation of Gel-OCS/MBGN hydrogels. Fig. 2 shows a schematic illustration of the interactions in Gel-OCS/MBGN hydrogels. It is known that an alkaline pH of the medium could enhance the reaction of Schiff's base formation [34]. Fig. S3 shows the typical pH change in PBS induced by the presence of Gel-OCS/MBGN hydrogels (15%). The hybrid hydrogel could rapidly increase the medium pH towards a more alkaline environment due to the released alkaline ions (in this context Ca^2+^) from MBGNs. The alkalinity of the medium should be greater with the concentration of MBGNs incorporated increasing, which could thus promote the Schiff's base reaction and accelerate the gelation. Also, the surface of MBGNs could be hydrated to form silanol groups (Si–OH) in an aqueous medium that could interact with the amino groups and carboxyl groups on Gel-OCS to form hydrogen bonding, which could also actively promote the crosslinking [19,34]. Additionally, the released Ca^2+^ might commence ionic gelation of OCS [35]. Therefore, it could be concluded that MBGNs facilitated the gelation of Gel-OCS/MBGN hydrogels probably due to the presence of these multi-interactions including both physical and covalent crosslinking mechanisms.

To be able to control the gelation behavior of hydrogels is important regarding their biomedical applications. For example, hydrogels developed for injectable or 3D printing technologies should have suitable and tailorable gelation time. Our results showed that the gelation time of Gel-OCS hydrogels could be controlled by tuning the concentration of MBGNs incorporated, which is of great interest to the applications of these hybrid hydrogels as injectable biomaterials or 3D printing inks for tissue regeneration. A thixotropic material can enable the injection of hydrogels using minimal force due to low viscosity. We thus evaluated the viscosity of the pre-crosslinked hydrogels at 37 °C (the measurement was performed as soon as the mixture of two components). Fig. 1f shows the shear-thinning properties of pre-crosslinked Gel-OCS hydrogels regardless of the concentration of MBGNs incorporated. The viscosity of the pre-crosslinked Gel-OCS hydrogels increased when the concentration of MBGNs added elevated but all decreased at higher shear rates. It should be noted that the pre-crosslinked Gel-OCS hydrogels could be smoothly extruded through the syringe and the addition of MBGNs for up to 15% (w/w) did not affect the injectability (Fig. 1g–h), which also evidenced the shear-thinning property of the hybrid hydrogel system. The good injectability allows the hydrogel to be applied as injectable biomaterials for treating bone defects with complex shapes, which brings convenience to minimally invasive therapy [36]. Meanwhile, due to the controllable gelation time of the hydrogels by the addition of MBGNs, the hybrid Gel-OCS/MBGN hydrogels could be crosslinked and shaped after the injection, which suggests their potential as gel inks for 3D printing applications.

### 3.3 MBGN incorporation enlarges pore size but not affecting the porous structure of Gel-OCS hydrogels

**Fig. 3:**
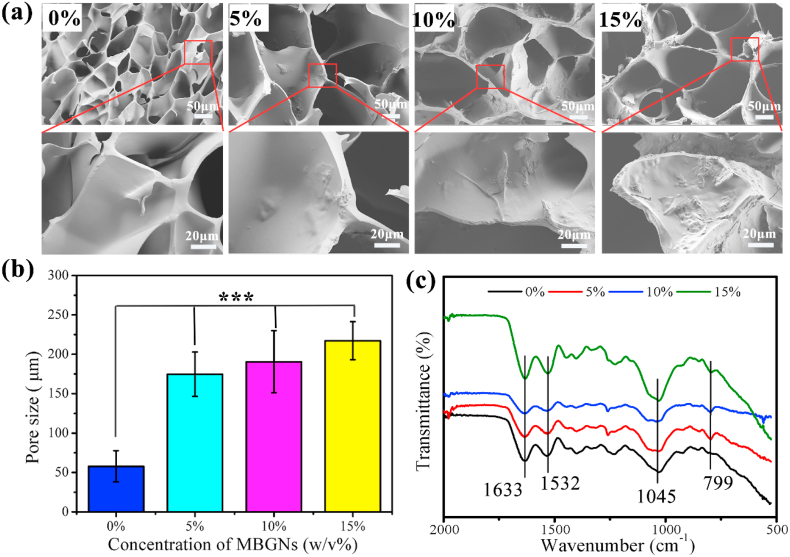
(a) SEM images of Gel-OCS/MBGN hydrogels with different concentrations of incorporated MBGNs. (b) Mean pore sizes of Gel-OCS/MBGN hydrogels. The pore size was determined by analyzing SEM images using Image J; (c) FTIR spectra of Gel-OCS/MBGN hydrogels.

The structure and pore size of the hydrogels were observed by SEM (Fig. 3a). The dried Gel-OCS hydrogel possessed a highly porous structure with an average pore size (~50 μm) (Fig. 3a), representing a typical porous structure for freeze-dried hydrogels [34]. The incorporation of MBGNs did not affect the porous structure of hydrogels. However, the pore size of the Gel-OCS/MBGN hydrogels was significantly larger than that of the Gel-OCS hydrogel (Fig. 3b). The average pore sizes of the dried hydrogels increased from ~50 to ~160, ~180 and ~217 μm when the concentration of added MBGNs reaching 5, 10 and 15%, respectively. Additionally, as the concentration of added MBGNs increased, the pore walls of the hydrogels became rough due to the presence of MBGNs on the surface. The pore size of the stent is 200–350 μm, which is most conducive to the growth of bone tissue.

A porous structure of scaffolds is important for successfully tissue regeneration as it allows migration and proliferation of cells as well as vascularization. It has been generally accepted that a pore size of ~100 μm should be sufficient to facilitate cell migration and tissue ingrowth [37,38]. Larger pore sizes have been reported to be able to facilitate vascularization [38]. However, the optimal pore size and porosity for scaffolds reported in the literature are usually valid for stiff scaffolds made of ceramics, glasses or metals. Freeze-dried polymeric hydrogels can naturally swell when they contact with an aqueous solution and exhibit relatively low stiffness, which may enlarge the pore sizes and allow for cell migration when they are applied in vitro and in vivo studies [39]. Also, the Gel-OCS hydrogel could degrade in an aqueous environment, thus leading to larger pores and voids over the incubation [19,40]. Therefore, hydrogel-based scaffolds with relatively small pore sizes (<100 μm) should also be sufficient for cell migration [41]. In this study, the mean pore size of Gel-OCS hydrogel was smaller than the reported optimal pore size for stiff scaffolds, but it can be anticipated that the pore sizes should be sufficient for further cell migration. Notably, the pore sizes of the hydrogels were larger than 100 μm after the incorporation of MBGNs, which met the criterion for optimal scaffolds for enabling cell migration, tissue ingrowth, and vascularization.

FTIR spectra results (Fig. 3c) confirmed the presence of MBGNs in the hybrid hydrogels. In all spectra, the characteristics bands of Gel and OCS could be observed. All the hydrogel demonstrated the characteristic bands reflecting its OCS structure, e.g., 947 cm^−1^ (C–O stretching), 1122 cm^−1^ (C–C stretching), and a shoulder starting at 1045 cm^−1^ (C–C and C–O stretching) [19]. The band located at 1532 cm^−1^ could be assigned to the N–H stretching vibration of amide II while the band located at 1633 cm^−1^ could be assigned to C–O and C–N stretching vibration of amide I in gelatin [34]. Compared to the spectrum of Gel-OCS hydrogel, two new bands located at 799 and 1100 cm^−1^ appeared after the incorporation of MBGNs, which could be assigned to Si–O–Si bending vibration and stretching vibration of silicate glasses, respectively, indicating the presence of MBGNs in the hybrid hydrogels [25]. As expected, the intensity of these two bands became stronger with elevating the concentration of MBGNs. FTIR results confirmed the presence of Gel, OCS, and MBGNs in the hybrid Gel-OCS/MBGN hydrogels.

### 3.4 Incorporation of MBGNs enhances mechanical properties of Gel-OCS hydrogels

**Fig. 4:**
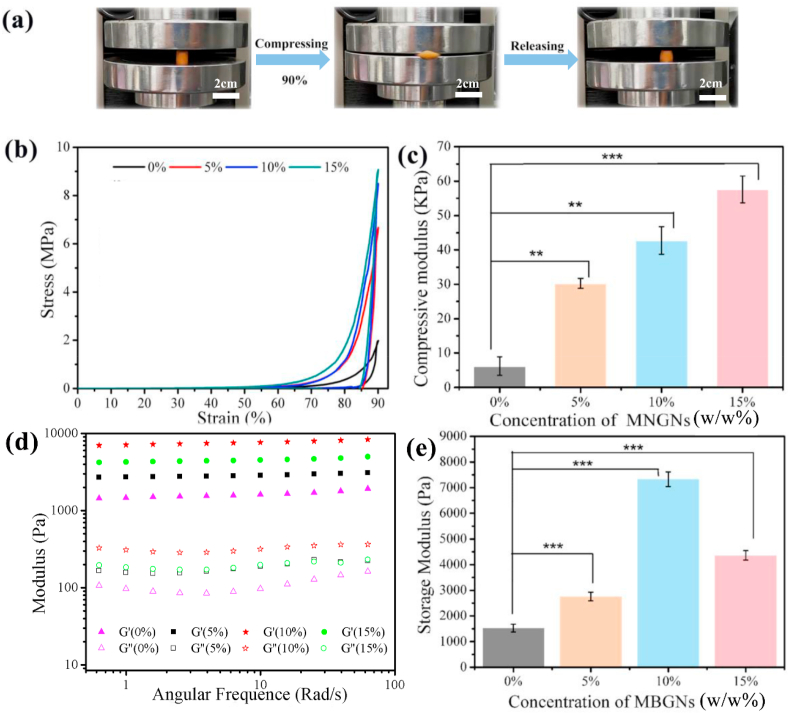
(a) Photographs of the process of compression test: Gel-OCS/MBGN (15%) hydrogels showing its ability to withstand large deformation under compression. (b) Typical compression stress-strain curves and (c) compressive modulus of the Gel-OCS/MBGN hydrogels at 25 °C. (d) Storage modulus (G′) and loss modulus (G’’) of the Gel-OCS/MBGN hydrogels under frequency sweep and (e) the storage modulus of the hydrogels at an angular frequency of 2 rad/s (*p < 0.05, **p < 0.01, ***p < 0.001).

To evaluate the effects of MBGN incorporation on the mechanical behavior of Gel-OCS hydrogels, the compression test was carried out on the samples. The compressive mechanical behavior is an important parameter for the hybrid hydrogels as this type of hydrogel is intended for bone regeneration/repair applications where the biomaterials mostly undergo compressive stress. As can be seen in Fig. 4a, the hybrid Gel-OCS/MBGN hydrogels (15% MBGNs one shown as an example) exhibited great elasticity and showed resilience to a certain extent after the compression test reaching 90% strain. Fig. 4b shows representative compressive stress-strain curves of all the hybrid hydrogels. The compression analysis of all the hydrogels showed the typical “J-shaped” stress-strain curves that resemble that of biological tissues (e.g., cartilage) [42]. The compressive strength values of the hydrogels at the maximum strain (~90%) were ~1.95, 6.58, 8.39, and 9.05 MPa for the hydrogels with 0, 5, 10, 15% of MBGNs, respectively. The compressive strength of Gel-OCS/MBGN hydrogels was located in the range of the reported compressive strength values of human cancellous bone (~0.15–13.7 MPa) [43]. Also, after the incorporation of MBGNs, the hydrogels still exhibited “J-shaped” compressive stress-strain curves. Moreover, the hydrogels maintained favorable elasticity after the incorporation of MBGNs, as indicated by their ability to withstand large deformation. Moreover, the compression modulus of the Gel-OCS hydrogel increased from ~6 KPa to ~30, ~43 and ~58 KPa when the concentrations of incorporated MBGNs were 5, 10, and 15%, respectively. An approximately 10-fold increase in compression modulus could be observed in the Gel-OCS/MBGN (15%) hydrogel in comparison to the Gel-OCS hydrogel (Fig. 4c). The effects of BG fillers in hydrogels on mechanical behavior have been reported in the literature [44,45]. For example, Zheng et al. [44] developed BG/GelMA based hydrogels for bone regeneration application. However, the compressive strength and modulus of composite hydrogels decreased after incorporation of BG, which was probably attributed to the relatively larger particle size (~300 nm) of BG and lack of interaction between BG and GelMA networks. In the present study, the introduction of MBGNs effectively enhanced the compressive strength and modulus of the hydrogels, which could be likely attributed to the enhanced crosslinking degree of the hydrogels induced by MBGNs. As mentioned above, MBGNs could facilitate the crosslinking by increasing the alkalinity, inducing hydrogen bonding and potential ionic gelation of OCS. Also, the small size of MBGNs contributed to an improved integration in the hydrogel matrices [24].

Besides the toughness, the stiffness of hydrogels also plays an important role in their successful bone regeneration application, as stiffness predominantly determines the stress distribution in biomaterial-bone systems [46]. Moreover, the stiffness of hydrogels can affect cell adhesion, morphology, cytoskeletal structure, and differentiation [47,48]. We thus evaluated the storage modulus and loss modulus of hydrogels by performing a frequency sweep rheological test at 37 °C. The results (Fig. 4d–e) showed that all experimental groups exhibited plateaued moduli, demonstrating the stability of all the hydrogels at physiological temperature (37 °C). In addition, the storage modulus and loss modulus were enhanced with the increase of MBGN concentration in the hydrogels. The storage modulus of the hydrogels increased from 1.5 kPa to 2.8, 7.3, and 4.4 kPa when the incorporations of MBGNs were 5, 10, and 15%, respectively. The storage modulus and loss modulus of hydrogels reached the largest values when the concentration of added MBGNs was 10%, but both moduli were slightly reduced when the concentration of MBGNs was further increased to 15%. All the hybrid Gel-OCS/MBGN still exhibited gel-like mechanical behavior after the incorporation of MBGNs. It has been known that hydrogels become stiffer when rigid inorganic fillers were incorporated [10]. Nanoparticles, such as silica nanoparticles, have also been used to regulate stiffness of hydrogel matrices to modulate cellular responses [49]. However, BGs have been rarely used as fillers to modulate hydrogel stiffness towards the control of cellular activities. Considering that BGs can release biologically active ions inducing specific cellular responses [50], synergetic effects of ions and stiffness on cellular activities should be considered when MBGNs are used as bioactive fillers in hydrogels, which will be the focus of our future studies. It also should be noted that the relatively high surface reactivity of MBGNs induces the release of ions from MBGNs that can interact with hydrogels (e.g., ionic crosslinking) changing the stiffness of hydrogel matrices.

### 3.5 Incorporation of MBGNs promotes mineralization and reduces degradation of hydrogels

**Fig. 5:**
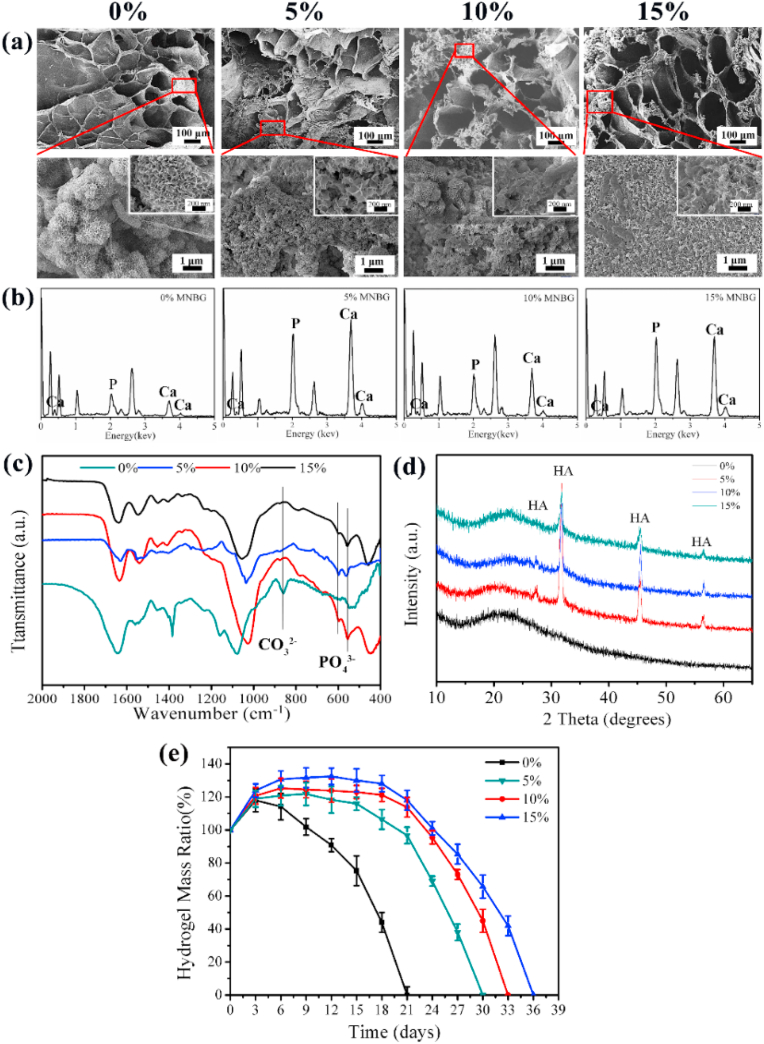
Evaluation of in vitro mineralization of hydrogels. (a) SEM images, (b) EDS spectra, (c) FTIR spectra, and (d) XRD patterns of Gel-OCS/MBGN hydrogels incorporating different concentrations of MBGNs after soaking in SBF for 7 days. (e) Mass reduction of the hydrogels after soaking in PBS at 37 °C.

Effective mineralization of hydrogels towards firmly bonding with bone tissues is also an important property of hydrogels intended for bone regeneration/repair applications [51]. Several strategies have been developed to mineralize polymeric hydrogels, such as the incorporation of enzymes able to catalyze the deposition of bone minerals (e.g., hydroxyapatite (HA)) or incorporation of bioactive fillers that can induce bone mineral formation [51]. BGs are well-known bioactive materials that can induce rapid surface mineralization. Therefore, besides modulating gelation behavior and enhancing mechanical properties, in the present study, MBGNs also acted as bioactive fillers to promote the mineralization of Gel-OCS hydrogels. It was observed that all hydrogels looked yellowish and exhibited a smooth surface before soaking in SBF (Fig. S4), but the hydrogels turned whitish after soaking in SBF for 7 days (Fig. S5), probably due to the formation of HA. SEM images (Fig. 5a) display the formed HA crystals on the surface of the hydrogels after 7 days of soaking in SBF. A cauliflower-like morphology could be observed on the surface of all hydrogels after soaking in SBF, which is the characteristic morphology of HA formed on bioactive surfaces in contact with physiological fluids [22]. As shown in SEM images, the coverage area of the formed HA layer on the surface of Gel-OCS hydrogel was smaller than that on the Gel-OCS/MBGN hydrogels. Moreover, denser layers of formed HA could be observed on the Gel-OCS/MBGN hydrogels incorporating a higher concentration of MBGNs. EDS results (Fig. 5b) showed that the intensity of Ca and P peaks became greater with the concentration of MBGNs increasing, suggesting a larger amount of formed HA on the hydrogels incorporating a larger concentration of MBGNs. FTIR results (Fig. 5c) also indicated the formation of HA on the hydrogels after soaking in SBF. Compared to the spectra of the hydrogels before soaking in SBF (Fig. 3c), two new bands located at ~560 and ~600 cm^−1^ were observed in the FTIR spectra of all hydrogels, which were assigned to the P–O bending vibrations in [PO_4_] tetrahedral related to apatite or other calcium orthophosphates [52,53]. The appearance of such bands indicated the formation of a crystalline HA phase on the hydrogels. In addition, the intensity of the bands was stronger on the Gel-OCS/MBGN hydrogels in comparison to that of the Gel-OCS hydrogel. XRD results (Fig. 5d) confirmed the formation of crystalline HA on the hydrogels after soaking in SBF for 7 days. Diffraction peaks located at 2θ = 26° (002), 32° (211), 39° (310), 46° (222), and 53° (004) related to HA crystals (JCPD 84–1998) could be observed in the XRD patterns of Gel-OCS/MBGN hydrogels [53], confirming that the formed crystals were HA. However, no diffraction peaks could be observed in the XRD pattern of Gel-OCS hydrogel, even though the presence of HA crystals had been observed in SEM images and indicated in the FTIR spectrum, which could be attributed to a low amount of formed HA, being below the detection limit of XRD. Taken together, the present results indicated that the incorporation of MBGNs promoted the in vitro mineralization of Gel-OCS hydrogel.

Biopolymer based hydrogels used for bone regeneration applications could suffer from limited mineralization (formation of HA layer) that would affect their effective bonding with bone tissues [51]. It has been known that CS could initiate the formation of HA due to the negative charge sites from the sulfate group binding charged calcium and phosphate ions [5]. Mineralization could also take place on gelatin-based hydrogels by either introducing precursors of HA [54] or modifying the chemical structure of gelatin [55]. Our results showed that HA formation could be observed in Gel-OCS hydrogel within 7 days of immersion in SBF, indicating the apatite forming ability of Gel-OCS hydrogel. However, after the incorporation of MBGNs, the mineralization of the hydrogels was promoted through the ion exchange and dissolution of MBGNs [13]. Bioactive glass nanoparticles have been used as bioactive fillers to enhance the mineralization of chondroitin sulfate [19] hydrogels and gelatin coatings [56]. Similar to the findings in the current study, the presence of BGNs significantly enhanced the formation of HA as well as promoting mechanical properties.

All the hydrogels still maintained the porous structure after 7 days of immersion in SBF (Fig. 5a). Notably, the pore size of Gel-OCS hydrogel was enlarged after the soaking in SBF, which could be attributed to the degradation of the hydrogel [34]. However, the pore sizes of Gel-OCS/MBGN hydrogels were not significantly changed, perhaps due to their higher degree of crosslinking leading to higher stability of these hydrogels. Fig. 5e shows the mass changes of the hydrogels after soaking in PBS (pH = 7.4) at 37 °C. As can be seen, the Gel-OCS hydrogel was degradable in physiological fluids and its completed degradation was observed after 21 days in PBS. After the incorporation of MBGNs, the degradation of the hydrogels was reduced. The times for the completed degradation were extended to ~30, 33 and 36 days for the Gel-OCS hydrogels incorporating 5, 10, and 15% MBGNs, respectively, which was attributed to enhanced crosslinking in the presence of MBGNs (Fig. 2). Biomaterials intended for tissue regeneration should be degradable in physiological fluids over time so that tissues are allowed for ingrowth. Both Gel and OCS are biodegradable biopolymers under physiological conditions. The degradation rate of Gel-OCS hydrogels could be modulated by controlling the Gel/OCS ratio and the oxidation degree of OCS [57]. Our results showed that the incorporation of MBGNs could also modulate the degradation of Gel-OCS hydrogels. However, comprehensive studies intended to understand the degradation behavior of these hybrid hydrogels were not carried out, which will be the focus of future studies.

### 3.6 Presence of MBGNs enhances proliferation of BMSCs

**Fig. 6:**
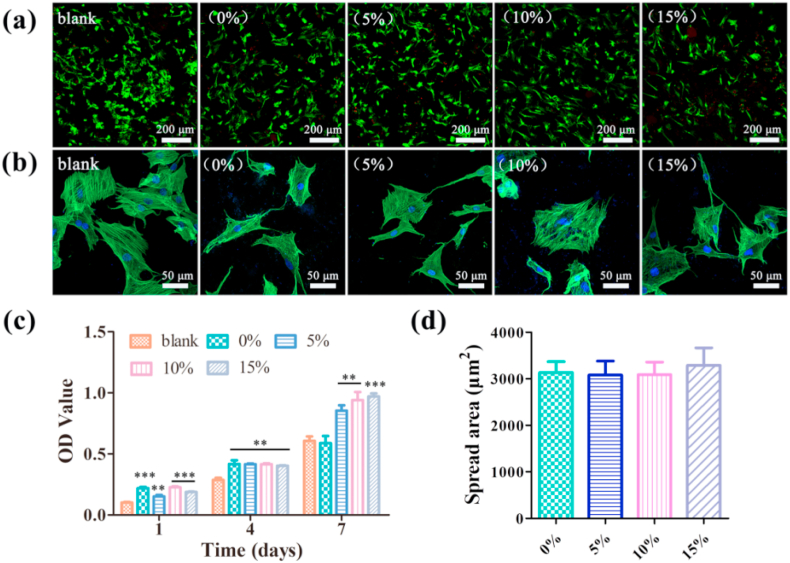
Adhesion and proliferation of BMSCs on the hydrogels. (a) Live-dead staining of cells cultured on the hydrogel after 24 h. (b) Cytoskeleton staining photos of cells cultured on the hydrogel after 48 h. (c) CCK-8 assay results of BMSCs cultured on the hydrogels after 1 day, 4 and 7 days. (d) Quantitative analysis of the cell spreading area. (n = 3, **p < 0.01, and ***p < 0.001).

The cytotoxicity of the hybrid hydrogels was examined by culturing BMSCs on the surface of the hydrogels. Cell viability was assessed by live/dead staining after 24 h of culture (Fig. 6a). A large number of living cells (green fluorescence) on all the hydrogels and only a few dead cells (red fluorescence) could be observed. The incorporation of MBGNs in the Gel-OCS hydrogels did not significantly reduce the number of visible living cells in comparison to that on the Gel-OCS hydrogels, indicating the non-cytotoxicity of all hydrogels. After 48 h of culture, the cytoplasm and nucleus of the cells on the hydrogels were stained. The images show (Fig. 6b) the cells spread well on the Gel-OCS hydrogel surfaces, suggesting the healthy status of the cells. Simultaneously, the proliferation of BMSCs on the hydrogels was quantitatively analyzed by the CCK-8 assay. Fig. 6c shows the OD values of the experiment groups, which showed that no significant difference could be observed between the Gel-OCS and Gel-OCS/MBGN hydrogels on day 1 and day 4. On day 7, a significant increase in OD values was observed in the hydrogels with MBGNs incorporated in comparison to the blank and the Gel-OCS hydrogel. A slightly higher OD value could be found in the Gel-OCS/MBGN hydrogels with a higher concentration of MBGN. The results indicate that the presence of MBGNs in the Gel-OCS hydrogel could promote BMSCs proliferation. The quantitative cell area analysis shows that the cell spreading on the hydrogels was not significantly changed after the incorporation of MBGNs, indicating the non-cytotoxicity of Gel-OSC/MBGN hydrogels (Fig. 6d)

### 3.7 Presence of MBGNs enhances osteogenic differentiation of BMSCs grown on Gel-OCS/MBGN hydrogel surfaces

**Fig. 7:**
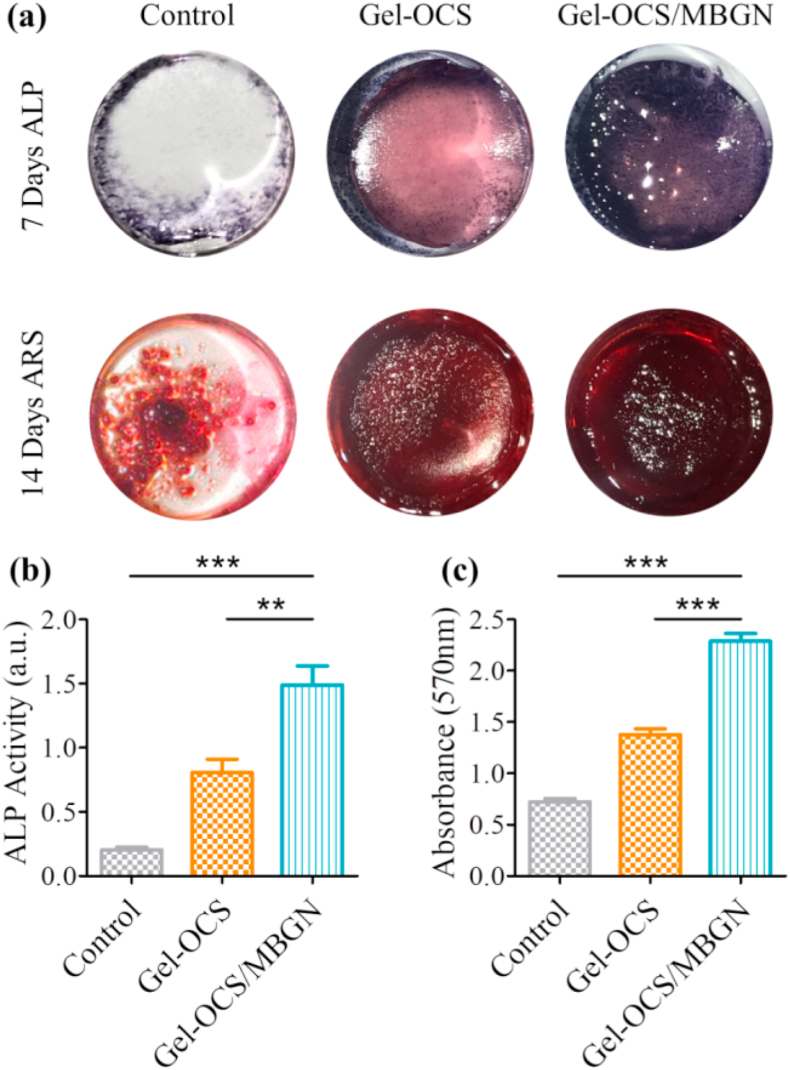
In vitro ALP expression and calcium biomineralization of BMSCs cultured on the hydrogels. (a) ALP staining of BMSCs on day 7 and Alizarin red S staining on day 14. (b) Quantitative ALP activity of BMSCs. c) Alizarin Red S stained mineral layer. (n = 3, **p < 0.01, and ***p < 0.001).

To evaluate the effects of incorporated MBGNs on the osteogenic differentiation of BMSCs, the activity of the early osteogenic marker alkaline phosphatase (ALP) and the mineralization in BMSCs were measured after 7 and 14 days of culture, respectively. The ALP staining results (Fig. 7a) showed that the ALP-positive areas incubated on the Gel-OCS hydrogel were visibly larger than that of the control group on day 7. The osteogenic potential of both Gel and CS have been shown in the literature [5,58,59], which could explain the enhanced ALP activity induced by the Gel-OCS hydrogel. Notably, the ALP positive areas were apparently increased when MBGNs were incorporated into the Gel-OCS hydrogel, indicating the further osteogenic differentiation of BMSCs. Alizarin red staining as a late osteogenic marker was used to measure mineralization. The results showed that the abundant mineralization nodules of Gel-OCS/MBGNs were dramatically intensified as MBGNs were incorporated (Fig. 7a). When the ALP and Alizarin red stains on the samples were quantified, the Gel-OCS/MBGNs showed the highest values and significant changes over other groups (Fig. 7b and c).

**Fig. 8:**
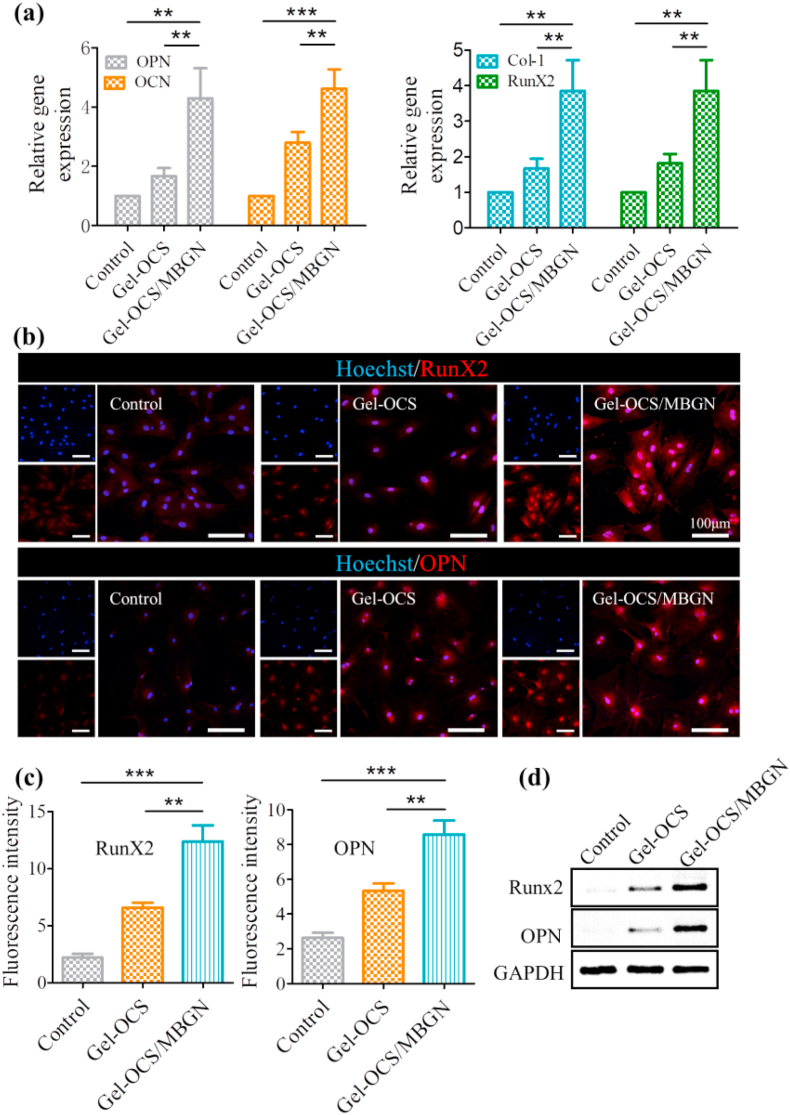
Osteoblast-related gene and protein expressions of BMSCs cultured on the hydrogel surfaces on day 14. (a) RT-PCR analysis of osteogenic-related genes encoding OPN, OCN, RunX2, and Col-1. (b) Representative immunofluorescent images of osteogenic-associated proteins RunX2 and OPN in different groups. RunX2 and OPN are marked by red fluorescence, and the cell nuclei were dyed blue by the Hoechst. (c) Quantitative RunX-2 and OPN fluorescence intensity. (d) Protein expression of RunX2 in BMSCs with GAPDH as a reference. (n = 3, **p < 0.01, and ***p < 0.001).

Osteogenic gene expressions including OPN, osteocalcin (OCN), RunX-2, and collagen I (Col-I) were further evaluated to investigate the osteogenic differentiation of BMSCs. All the selected genes were upregulated after incubation on Gel-OCS/MBGNs as compared to those cultured on control plate and Gel-OCS groups (Fig. 8a). The expression pattern was similar in immunofluorescence staining; the highest osteogenic-associated proteins RunX-2 and OPN fluorescence intensity were detected in the Gel-OCS/MBGNs group. In addition, quantitative analysis of fluorescence intensity showed a similar tendency (Fig. 8c). Consistently, the Western blot results in Fig. 8d also indicated that the Gel-OCS/MBGNs group was more favorable for protein expression of RunX2 and OPN than the others. All these results demonstrate that the presence of MBGNs in Gel-OCS hydrogels has the ability to promote BMSC osteogenic differentiation. The hybrid Gel-OCS/MBGN hydrogels can thus be used in applications concerning bone regeneration.

### 3.8 Presence of MBGNs promotes bone regeneration in vivo

**Fig. 9:**
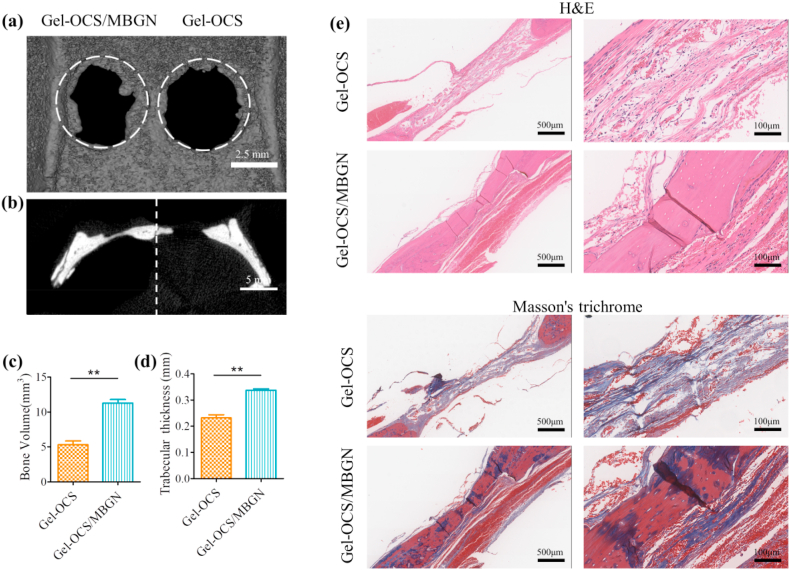
In vivo bone regeneration after 6 weeks of implantation of the hydrogels. (a) Representative micro-CT 3D reconstructed images and (b) sagittal view images of critical-sized rat calvarial defects. Quantitative analysis of (c) bone volume and (d) trabecular thickness (**p < 0.01). (e) H&E− and Masson's trichrome staining of histological sections of calvarial decalcified sections after hydrogel implantation.

To evaluate the in vivo bone regenerative capacity of Gel-OCS and Gel-OCS/MBGNs hydrogels, the two hydrogels were directly injected into freshly formed rat cranial defects. Bone tissue regeneration was studied using micro-CT and histological examinations. After 6 weeks of implantation of the hydrogels, new autologous bone tissue appeared around the margin of the critical-sized defect in the Gel-OCS/MBGNs group, while the Gel-OCS group showed limited new bone formation (Fig. 9a). Sagittal crosssection images confirmed that the consecutive regenerated bone was formed in the defect in the Gel-OCS/MBGNs group, compared with the Gel-OCS group (Fig. 9b). As expected, the quantitative micro-CT analysis showed that the Gel-OCS/MBGNs group presented a significantly higher regenerated bone volume (Fig. 9c) and thicker trabecular bone (Fig. 9d) than the Gel-OCS group. The results of micro-CT and quantitative analysis revealed significantly better bone regenerative capacity in the MBGNs-containing Gel-OCS/MBGNs group in comparison to the Gel-OCS group, suggesting that Gel-OCS combined with MBGNs could lead to faster and more efficient healing of bone defects.

Hematoxylin & Eosin (H&E) and Masson's trichrome further confirmed that the Gel-OCS/MBGNs hydrogel facilitated improved new bone regeneration. After 6 weeks, the formation of nearly complete mature osseous tissue along the junction of the defects was clearly observed for the Gel-OCS/MBGNs group. However, in the Gel-OCS group, small amounts of regenerating new bone tissue and less bone formation near the margin of the original defect were observed, and no complete and continuous osseous closure appeared (Fig. 9e).

Thus, the above results from the micro-CT and histology analyses suggest that the Gel-OCS/MBGNs hydrogel could significantly and efficiently promote bone defect regeneration in vivo, compared to Gel-CS hydrogel. The regenerative capacity of the hybrid composite hydrogel material can be attributed to the presence of MBGNs, which are effective in promoting osteogenic differentiation. The in vivo results are consistent with the in vitro results discussed previously. The incorporation of MBGNs showed markedly increased osteogenic differentiation capacity, which facilitated the growth of new bone in vivo. Therefore, Gel-OCS/MBGNs hydrogel can be considered a promising bone regeneration biomaterial.

## 4 Conclusions

In this study, injectable self-crosslinking hybrid Gel-OCS/MBGN hydrogels were developed for bone regeneration applications. The incorporation of MBGNs promoted crosslinking and accelerated the gelation process. The gelation time of the hybrid hydrogels could be tuned by adjusting the concentration of MBGNs added. The gelation time decreased with increasing concentration of MBGNs incorporated. The presence of MBGNs in the hydrogel significantly enhanced the storage modulus and compressive strength. Moreover, the injectability of the hydrogels was not significantly reduced after the incorporation of MBGNs. Also, Gel-OCS/MBGN hydrogels significantly promoted the proliferation and osteogenic differentiation of BMSCs in vitro and showed effective bone regeneration in vivo compared with Gel-CS hydrogels. The hybrid hydrogels show thus great potential as injectable biomaterials or scaffolds for bone regeneration/repair applications given their tunable degradation and gelation behavior as well as the favorable mechanical behavior and osteogenic activity.

## CRediT authorship contribution statement

**Lei Zhou:** Writing - original draft, designed and performed experiments and participated in the writing of the article. **Lei Fan:** designed and performed the in vivo animal tests, provided suggestions for the experimental design. **Feng-Miao Zhang:** Writing - original draft, designed and performed experiments and participated in the writing of the article. **Yuhe Jiang:** provided suggestions for the experimental design. **Min Cai:** designed and performed the in vivo animal tests. **Cong Dai:** provided suggestions for the experimental design. **Yi-An Luo:** conducted the mineralization experiment. **Ling-Jie Tu:** Writing - original draft, provided suggestions for experimental protocols and article writing. **Zheng-Nan Zhou:** Writing - original draft, provided suggestions for experimental protocols and article writing. **Xiao-Jun Li:** Writing - original draft, provided suggestions for experimental protocols and article writing. **Cheng-Yun Ning:** Supervision, supervised the work and provided constructive comments. **Kai Zheng:** Writing - original draft, designed experiments and contributed to data analysis and manuscript writing. **Aldo R. Boccaccini:** Supervision, supervised the work and provided constructive comments. **Guo-Xin Tan:** Supervision, supervised the work and provided constructive comments.

## Declaration of competing interest

The authors declare no conflict of interest.
