# Supplementary material for: Hybrid gelatin/oxidized chondroitin sulfate hydrogels incorporating bioactive glass nanoparticles with enhanced mechanical properties, mineralization, and osteogenic differentiation
Source: Bioact Mater. 2020 Oct 6;6(3):890–904. doi: 10.1016/j.bioactmat.2020.09.012 (PMC7548431; doi:10.1016/j.bioactmat.2020.09.012)
Supplement: supplementary data [file mmc1.docx]

# **Supplementary information**

# **Hybrid gelatin/oxidized chondroitin sulfate hydrogels incorporating bioactive glass nanoparticles with enhanced mechanical properties, mineralization, and osteogenic differentiation**

Lei Zhou^1, 3,^ ^#^, Lei Fan^3, #^, Feng-Miao Zhang^1^^,^ ^#^, Cong Dai^1^, Yuhe Jiang^4^, Min Cai^5^, Yi-An Luo^1^, Ling-Jie Tu^1^, Zheng-Nan Zhou^1^, Xiao-Jun Li^1^, Cheng-Yun Ning^3^, Kai Zheng^2^*, Aldo R. Boccaccini^2^*, Guo-Xin Tan^1^*

^1^Institute of Chemical Engineering and Light Industry, Guangdong University of Technology, Guangzhou, 510006, China

^2^Institute of Biomaterials, Department of Materials Science and Engineering, University of Erlangen-Nuremberg, Cauerstrasse 6, 91058 Erlangen, Germany

^3^School of Materials Science and Engineering, South China University of Technology, Guangzhou, 510641, China

^4^College of Arts and Sciences, Stony Brook University, 100 Nicolls Road, Stony Brook, New York 11794, United States

^5^Department of Prosthodontics, Hospital of Stomatology, Guanghua School of Stomatology, Sun Yat-sen University, Guangzhou, 510055, China

^#^ These authors contributed equally to this work

^*^Corresponding authors: K. Z. (kai.zheng@fau.de), A. R. B. ([aldo.boccaccini@ww.uni-erlangen.de](mailto:aldo.boccaccini@ww.uni-erlangen.de)); G.X. T. (tanguoxin@126.com)


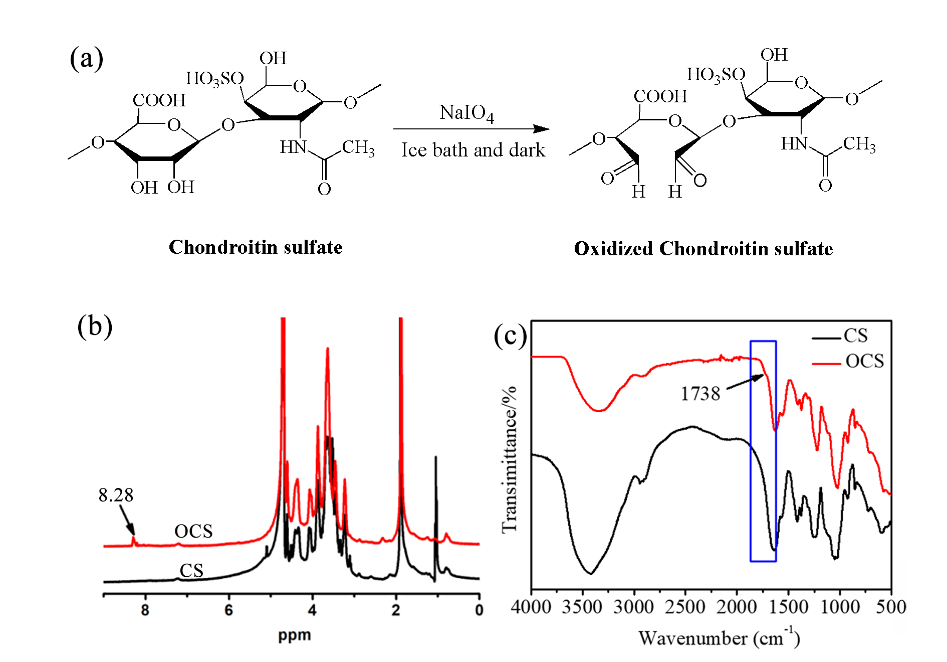


**Figure S1** Characterization of OCS. (a) OCS synthesis equation, (b) ^1^H-NMR and (c) FTIR characterization


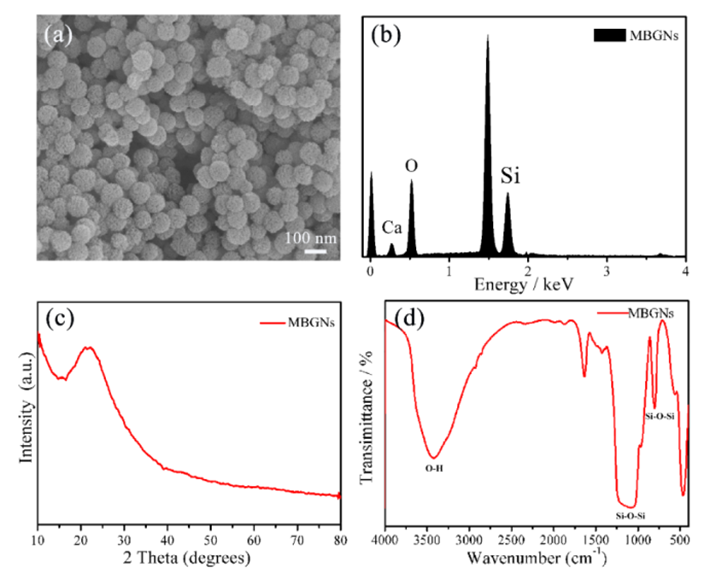


**Figure S2**. Characterization of MBGNs. (a) SEM image, (b) EDS spectrum, (c) XRD pattern and (d) FTIR spectrum of MBGNs


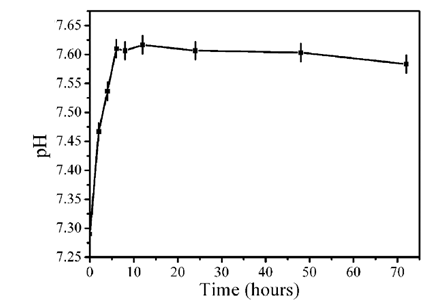


**Figure S3** Representative pH values change overtime in PBS induced by Gel-OCS/MBGN (10%)


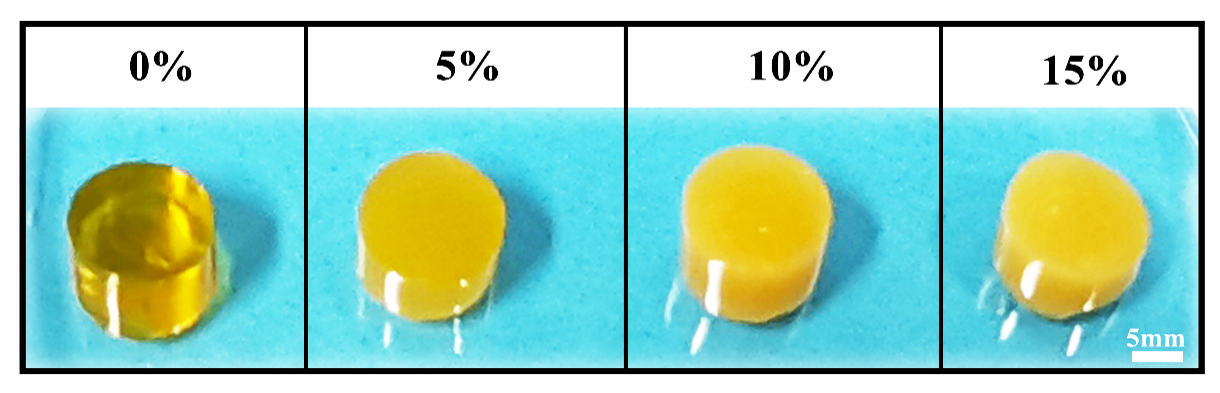


**Figure S4** Optical images of the as-prepared Gel-OCS/MBGN hydrogels with different concentrations of MBGNs incorporated (0, 5, 10, and 15%).


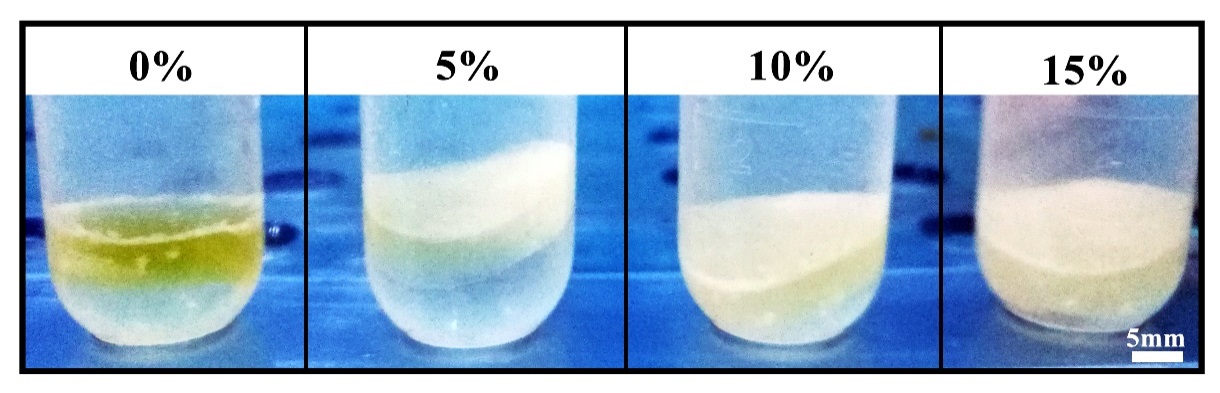


**Figure S5** Optical images of the Gel-OCS/MBGN hydrogels after immersion in SBF for 7 days

| **Table S1** Primer sequences of each gene | | |
| --- | --- | --- |
| Gene | Forward | Reverse |
| RunX2 | TCTTCCCAAAGCCAGAGCG | TGCCATTCGAGGTGGTCG |
| Col-1 | CTGCCCAGAAGAATATGTATCACC | GAAGCAAAGTTTCCTCCAAGACC |
| OPN | AAGCCTGACCCATCTCAGAA | GCAACTGGGATGACCTTGAT |
| OCN | AAACATGGCAAGGTGTGTGA | AGGTGACCAGGACGTTTTTG |
